# Validation of questionnaire on the Spiritual Needs Assessment for Patients (SNAP) questionnaire in Brazilian Portuguese

**DOI:** 10.3332/ecancer.2016.694

**Published:** 2016-11-22

**Authors:** Diego de Araujo Toloi, Deise Uema, Felipe Matsushita, Paulo Antonio da Silva Andrade, Tiago Pugliese Branco, Fabiana Tomie Becker de Carvalho Chino, Raquel Bezerra Guerra, Túlio Eduardo Flesch Pfiffer, Toshio Chiba, Rodrigo Santa Cruz Guindalini, Daniel P Sulmasy, Rachel P Riechelmann

**Affiliations:** 1 Instituto do Câncer do Estado de São Paulo, 01246-000, Brazil; 2 Faculty of Medicine of São Paulo University, 01246-903, Brazil; 3 School of Medicine and Divinity School, Chicago University, IL 60637, USA

**Keywords:** cancer, spirituality

## Abstract

**Objectives:**

Spirituality is related to the care and the quality of life of cancer patients. Thus, it is very important to assess their needs. The objective of this study was the translation and cultural adjustment of the Spiritual Needs Assessment for Patients (SNAP) questionnaire to the Brazilian Portuguese language.

**Methodology:**

The translation and cultural adjustment of the SNAP questionnaire involved six stages: backtranslation, revision of backtranslation, translation to the original language and adjustments, pre-test on ten patients, and test and retest with 30 patients after three weeks. Adult patients, with a solid tumour and literate with a minimum of four years schooling were included. For analysis and consistency we used the calculation of the Cronbach alpha coefficient and the Pearson linear correlation.

**Results:**

The final questionnaire had some language and content adjustments compared to the original version in English. The correlation analysis of each item with the total score of the questionnaire showed coefficients above 0.99. The calculation of the Cronbach alpha coefficient was 0.9. The calculation of the Pearson linear correlation with the test and retest of the questionnaire was equal to 0.95.

**Conclusion:**

The SNAP questionnaire translated into Brazilian Portuguese is adequately reliable and consistent. This instrument allows adequate access to spiritual needs and can help patient care.

## Introduction

Spirituality can be defined as the relationship of a person with what he/she considers to be transcendent and can happen in different ways, one of them being religion [1]. The relationship of spirituality with quality of life is described in the literature [2], with studies showing the relationship between spiritual well-being and better indexes of quality of life [3]. Furthermore, it is believed that spiritual demands that are not addressed can compromise the care of the patient. This is based on studies that have shown that many consider addressing spirituality to be important for their treatment [4, 5].

A systematic review published in 2011 reports 35 instruments validated for the assessment of spirituality and four with the concept of need of spirituality: *Spiritual Need Inventory* (SNI) - assessed for patients near the end of life, *Spiritual Interests Related to Illness Tool* (SpIRIT) - developed for patients and family members, *Spiritual Needs Scale* (SNS) - Korean questionnaire with 26 items validated by Yong *et al* [6], and the *Spiritual Needs Questionnaire* (SpQN) - developed for patients with chronic diseases [7]. Another systematic revision of spirituality questionnaires in the Portuguese language shows the existence of 20 instruments, 15 of which are translated from other languages, but without the different instruments of spiritual need [8].

The assessment of the spiritual needs of patients is important to provide integral care. The *Spiritual Needs Assessment For Patients* (SNAP) questionnaire was developed and validated for the English language in 2012, The objective was for assessing the spiritual need of patients with oncologic and haematologic diseases based on psychosocial, spiritual and religious subscales, and covers aspects of the cognitive, behavioural and affective spheres [9]. Nevertheless, for these studies to be used for patients whose native language is Portuguese, the translation and cultural adjustment of this instrument is required.

The objective of this study was to document the translation and cultural adjustment of the SNAP questionnaire to the Brazilian Portuguese language: Assessment of Spiritual Need for Patients.

## Methods

SNAP assesses the spiritual need of patients based on three subscales: psychosocial (five items), spiritual (13 items), and religious (five items)—containing closed questions that assess the need of the patients in these items. The alternatives for the replies are graded from 1–4, the total score can vary from 23–92, and higher values reflect more spiritual needs [9].

The validity of an instrument is the degree in which the measurements are able to assess what the instrument intends to ascertain, so the translation and cultural adaptation of the questionnaire between different cultures and languages is important to analyse if the translated instrument adequately reflects the items of the original version [10].

The translation and validation of the SNAP questionnaire involved six stages. The first stage was the translation, where the SNAP questionnaire was independently translated into Portuguese by two Brazilian physicians (T.E.F.P. and F.M.) who are familiar with the English language and with the theme of spirituality. The second was a revision of the translated items to assess coherence, and was carried out by an independent group of eight professionals in the health area (D.A.T., D.U., P.A.S.A., T.P.B., F.T.B.C.C., R.B.G., T.C., R.S.P.R.). The third stage was the backtranslation, where this new revised version was translated again into English by a qualified translator. Here the incoherences were identified and readjusted into Portuguese, thus obtaining the final version of the questionnaire (D.A.T., R.S.G., D. S., R.S.P.R.). The fourth stage was the pre-test, i.e. the final version was applied on a sample of ten patients [11, 12] according to the eligibility criteria, as an interview, in order to perform interactive adjustments and better refine the translated questions and replies (D.A.T., F.M., D.S., R.S.P.R.). At this stage, the patients gave their opinion and challenges with the SNAP questions to let know their understanding to this.

The fifth and sixth stages correspond to the assessment of the consistency itself: the questionnaire in its final translated form was applied to another 30 eligible patients and then re-applied to them after three weeks (admitting a window of about two days) (D.A.T., D.U., F.M., P.A.S.A.). Around 30 patients were chosen for convenience and for being similar to the one used in studies of cultural adaptation [13–15].

The criteria for patient inclusion were age equal to or above 18 years with solid tumour in treatment with curative intention, or with palliative chemotherapy, or with exclusive palliative care, literate with minimum schooling of four years and having what the researcher deems to be the ability to adequately comprehend the questions. The exclusion criteria were a performance status that might compromise adequate participation at the criterion of the investigator, difficulty in spontaneous communication in Portuguese for any reason, disoriented and confused patients, and patients in the first two months of monitoring.

The following features of the participating patients were collected: age, gender, auto-referred race, civil status, religion, neoplasia type, and treatment intent.

The statistical analysis assessed the reliability of the translated instrument. The reliability of the instrument is the degree in which its measuring is free from measurement errors, which is to say it assesses by how much points the patients do not change with repeated measurements in various conditions [10]. Reliability has the property of internal consistency [10]. Internal consistency is the degree of interrelation among the items that compose the instrument, and was assessed by calculating the Cronbach alpha coefficient of the entire questionnaire as well as of each subscale. The Cronbach alpha coefficient is a measurement of the uniformity of the items [16]. The reliability of the instrument was also assessed for congruence by analysing the item-total correlation.

The test-retest assesses the consistency of the questionnaire. This being defined as a measurement of the reproductibility of the instrument, i.e., the ability to reflect constant results in an interval of time for the same population. Pearson linear correlation was used for analysis [17].

The study was performed at the Instituto do Câncer do Estado de São Paulo, one of the largest Latin American cancer treatment centres. The study was approved by the Committee of Ethics and Clinical Research of the Faculty of Medicine of São Paulo University (procedure number 129/13) and informed consent was obtained from all the patients.

## Results

Forty patients participated, ten in the pre-test stage of the translation and 30 in the consistency assessment stages. All the patients who were offered the study agreed to participate. The features of the participants are described in Table 1.

The translation process required adaptation of the original item referring to dealing with the concept of death and death itself (item 14) since no distinction was observed between the expressions 'death' and 'die' during the interviews performed in stage four (pre-test). Items 22 and 23 with terms referring to practices and books raised questions from the participants and received additions with 'pass' in item 22 and 'The gospel according to spiritism' in item 23. The final version of the translated and validated questionnaire is in Addendum 1.

**Figure 1.**
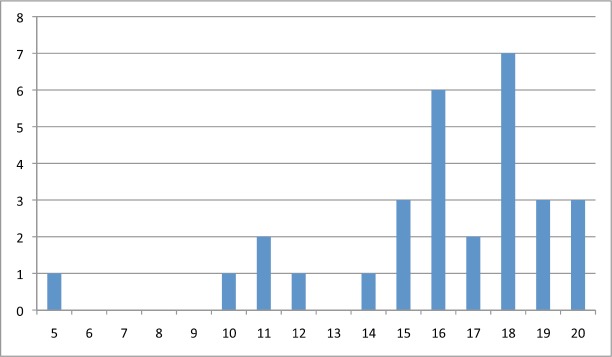
Distribution of psychosocial subscale scores.

**Figure 2.**
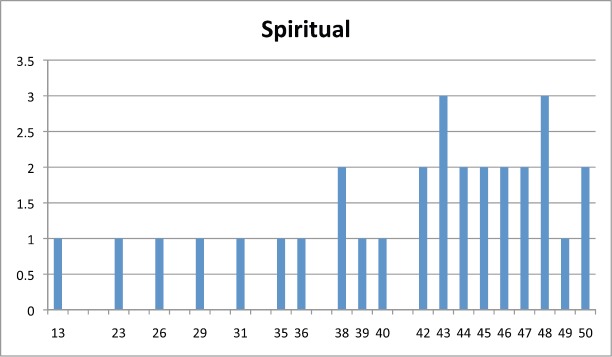
Distribution of spiritual subscale scores.

**Figure 3.**
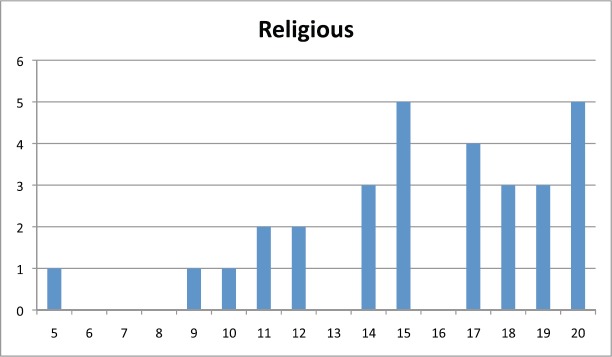
Distribution of religious subscale scores.

Table 2 shows the average score for each question per subscale and Figures 1, 2, and 3 show the distribution of scores per subscale of the 30 participants in the fifth stage.

The time required to fill in the questionnaire was from 1 to 2 minutes.

The correlation analysis of each item with the total score of the questionnaire showed internal validity with coefficients above 0.99 for all questions (Table 3).

The Cronbach alpha coefficient was 0.9, with the corresponding calculations for the psychosocial, spiritual, and religious subscales: 0.72, 0.87, and 0.79 (Table 4).

The calculation of the Pearson linear correlation with the test and retest of the questionnaire was equal to 0.95 (IC 0.9–0.98).

## Discussion

This study brings the translation and cultural adjustment of the SNAP questionnaire to the Brazilian Portuguese language, presenting excellent internal validity, reliability, and consistency.

Spirituality can be assessed in different manners: current status, well-being, confronting, and spiritual need; and in categories: cognitive (attitudes and beliefs), behavioural (practices), and affective (associated feelings) [7]. The assessment of spiritual need has no instruments in the Portuguese language, making studies of spirituality difficult in our environment. SNAP is a questionnaire that covers spiritual need in the three described categories [9].

Among the participants, we had a majority of patients in systemic treatment with palliative intent and a large representation among the various types of neoplasia (Tables 1 and 2). This was important to understand and to adjust the questionnaire per different groups.

Comparing the original data of the development and validation of the questionnaire in the English language [9], the data of the cultural adjustment to the Mandarin language [15], and those of this cultural adjustment, the Brazilian spiritual need seems to be greater than the others, with higher average scores in the three subscales respectively: psychosocial 12.1, 13.7, and 16.03, spiritual 30.1, 28.5, and 40.57, and religious 9.5, 8.3, and 15.53 [9, 15]. The reliability assessment between our study and those mentioned above was similar. The internal consistency assessment with the Cronbach alpha coefficient for the validation studies in English, Mandarin, and Portuguese was respectively, 0.95, 0.89, and 0.9 [9, 15].

Almost all the clinical studies of phase III in oncology are multicentric and many are international. The quality of life assessment is common in these studies, especially those that register new medications. In this context, due to the variety of countries and languages of the participating patients, translation of the instruments into each language is required. As it is impossible to have validation for all languages, many studies accept the translation and back-translation as adequate for instruments already broadly validated. Although the cultural adjustment of questionnaires to different languages is a complex, lengthy process, we consider it necessary, especially for 'new' questionnaires. We also consider the cultural adjustment of questionnaires to other languages important when their score is the primary outcome.

For example, in our study, adjustments were required for including the item of the 'gospel according to spiritism' book and 'pass', as kardecist spiritism is a common practice in Brazil. Still as regards items 22 and 23, we verified that the participants were curious about practices being different from those stated, creating the hypothesis that the instruments that address spirituality, with the possibility of promoting questioning, can also be a kind of intervention. These stimulate the participants to think and reflect on their own spirituality besides measurement of demand.

A limitation of our study is that we included patients from the São Paulo region. Brazil is a country with continental proportions and different cultures are common in the various regions. Nevertheless, the patients being treated at the Institute come from various states which reduces the risk of biased selection in our sample. Furthermore, the Portuguese language is spoken in the whole country. However, we cannot exclude the possibility that cultural language adjustments would have been required if the study had been performed in other states. Another limitation is the absence of atheist and illiterate patients, a variable that can involve the external validity of the instrument. The number of patients is reduced, but it is similar to other cultural adjustment studies too [13, 13, 15]. Although the study was performed with oncologic parties, respecting the validity of the content of the original instrument, we think that it can be used in general with patients with other diseases. Another limitation is that due to similar instruments that assess the spiritual need of patients in native Portuguese language, the concurrent validity and divergent validity were not performed.

## Conclusion

There has been little study of spirituality in oncology [3]. The cultural adjustment of the SNAP questionnaire to the Brazilian Portuguese language showed high levels of reliability and consistency. Therefore, we find this offers numerous study opportunities for assessing spiritual needs in various oncologic scenarios, besides intervention studies, with the objective of providing integral care to cancer patients. Studies with a larger number of patients are required to confirm the external validity of SNAP in Portuguese.

## Figures and Tables

**Table 1.** Demographic data for 4 (pre-test), 5, and 6-stage patients (adaptation of questionnaire).

Total N = 40		
Average Age		53.8 (25–83)
Gender		
	Male	40% (16)
	Female	60% (24)
Ethnicity		
	White	65% (26)
	African Descent	15% (6)
	Mixed Race	17.5% (7)
	Asiatic	2.5% (1)
Marital Status		
	Married	62.5% (25)
	Single	22.5% (9)
	Widowed	7.5% (3)
	Divorced	7.5% (3)
Religion		
	Catholic	52.5% (21)
	Protestant	32.5% (13)
	Belief in God, no religion	5% (2)
	Other Religions	5% (2)
	Spiritism	2.5% (1)
	Jehovah’s Witness	2.5% (1)
Neoplasia		
	Gastrointestinal	42.5% (17)
	Breast	22.5% (9)
	Sarcoma	12.5% (5)
	Melanoma	5% (2)
	Endocrine	5% (2)
	Head and Neck	5% (2)
	Prostate	2.5% (1)
	Lung	2.5% (1)
	Gynaecological	2.5% (1)
Treatment		
	Palliative in systemic treatment	72.5% (29)
	Curative	27.5% (11)

**Table 2.** Item Scores.

Items by subscale of need	Average
Psychosocial 1. Contact with other patients with similar illnesses? 2. Any relaxation or stress-relieving activity? 3. Learning to cope with feelings of sadness? 4. Aprender a lidar com sentimentos de tristeza? 5. Sharing your thoughts and feelings with people close to you? 6. Concerns you have with your family?	16.03 3.07 3.00 2.93 3.37 3.67
Spiritual 7. Finding meaning in your experience of the illness? 8. Finding hope? 9. Overcoming fear? 10. Meditation (personal) or praying? 11. Your relationship with God, or something beyond yourself? 12. Staying closer to a community that shares your spiritual beliefs? 13. Dealing with any suffering you may be going through? 14. The meaning and purpose of human life? 15. Dying? 16. Finding peace of mind? 17. Resolving old disputes, grievances, or resentment between family and friends? 18. Finding forgiveness? 19. Taking decisions about your medical treatment that are in accordance with your spiritual or religious beliefs?	40.57 3.43 3.40 3.23 3.47 3.53 3.13 3.10 3.33 2.23 3.40 2.33 3.40 2.87
Religious 20. Visits from a religious leader from your own religious community? 21. Visits from a hospital pastor/priest? 22. Visits from members of your religious community? 23. Any religious ritual such as chanting, lighting candles or incense, anointing, communion, prayer, or laying-on of hands? 24. Someone to bring you religious texts such as the Bible, the Gospel according to Spiritism, the Torah, the Qur’an (Koran), the Analects of Confucius or the Tibetan Book of the Dead?	15.53 3.27 3.00 3.37 2.83 3.07

**Table 3.** Item-total correlation analysis.

Question	Correlation
1	0.9977
2	0.9977
3	0.9978
4	0.9985
5	0.9988
6	0.9984
7	0.9985
8	0.9977
9	0.9989
10	0.9986
11	0.9984
12	0.9975
13	0.9986
14	0.9967
15	0.9985
16	0.9968
17	0.9976
18	0.9973
19	0.9978
20	0.9979
21	0.9978
22	0.9955
23	0.9972

**Table 4.** Cronbach’s alpha coefficient.

	α (confidence interval)
Total	0.9 (0.86–0.93)
Psychosocial Subscale	0.72 (0.6–0.8)
Spiritual Subscale	0.87 (0.82–0.9)
Religious Subscale	0.79 (0.7–0.85)

## ADDENDUM 1. SNAP questionnaire in Portuguese (English version available in [9]).

Por favor, escolha a opção que melhor descreve seu nível de necessidade a respeito de como você está lidando com sua doença. Você pode ter necessidades agora. Ou, pode ter necessidades mais tarde. Por favor, responda sobre qualquer necessidade que você tenha agora ou acha que pode ter mais tarde. Se você acha que nunca vai ter a necessidade, por favor marque “De maneira nenhuma”.

**Table:**

**O quanto você gostaria de ajuda para:**	**Muito**	**Um pouco**	**Não (muito)**	**De maneira nenhuma**
1. Entrar em contato com outros pacientes com doenças semelhantes?				
2. Alguma atividade de relaxamento ou para diminuição de estresse?				
3. Aprender a lidar com sentimentos de tristeza?				
4. Compartilhar seus pensamentos e sentimentos com pessoas próximas a você?				
5. Preocupações que você tem com sua família?				
6. Encontrar significado na sua experiência com a doença?				
7. Encontrar esperança?				
8. Superar medos?				
9. Meditação (pessoal) ou prática de orações?				
10. Seu relacionamento com Deus ou algo além de você?				
11. Ficar mais próximo de uma comunidade que compartilhe de suas crenças espirituais?				
12. Lidar com qualquer sofrimento que você esteja passando?				
*Por favor, responda sobre qualquer necessidade que você tenha agora ou acha que pode ter mais tarde.*				
**O quanto você gostaria de falar com alguém sobre:**	**Muito**	**Um pouco**	**Não (muito)**	**De maneira nenhuma**
13. O significado e propósito da vida humana?				
14. O morrer?				
15. Encontrar paz de espírito?				
16. Resolver disputas antigas, mágoas ou ressentimentos entre familiares ou amigos?				
17. Encontrar perdão?				
18. Tomar decisões sobre seu tratamento médico que estejam de acordo com suas crenças espirituais ou religiosas?				
**O quanto as seguintes situações seriam benéficas para você?**	**Muito**	**Um pouco**	**Não (muito)**	**De maneira nenhuma**
19. Visitas de um líder religioso da sua própria comunidade religiosa?				
20. Visitas de um pastor / padre do hospital?				
21. Visitas de membros de sua comunidade religiosa?				
22. Algum ritual religioso como cânticos, acender velas ou incensos, unção, comunhão ou oração ou passe?				
				
23. Alguém trazer para você textos espirituais como a Bíblia, Evangelho segundo o Espiritismo, Torah, Alcorão (Corão), Analectos de Confúcio ou O Livro Tibetano dos Mortos?				
